# The *Escherichia coli* Translation-Associated Heat Shock Protein YbeY Is Involved in rRNA Transcription Antitermination

**DOI:** 10.1371/journal.pone.0062297

**Published:** 2013-04-29

**Authors:** Maya Grinwald, Eliora Z. Ron

**Affiliations:** Department of Molecular Microbiology and Biotechnology, Life Sciences, Tel Aviv University, Tel Aviv, Israel; Kinki University School of Pharmaceutical Sciences, Japan

## Abstract

A new group of translation-associated heat shock genes has been recently identified. One of these novel genes is *ybeY* which is highly conserved in bacteria. In *Escherichia coli* the YbeY protein is important for efficient translation at all temperatures and is essential at high temperatures. Deletion mutants of *ybeY* are defective in protein translation, due to impaired 30 S ribosomal subunits. Here we provide evidence which tie YbeY to the transcription antitermination process. Thus, in *ybeY* deletion mutants transcription is significantly inhibited when the “*nut* like” sequences required for transcriptional antitermination are present, while if these sequences are removed transcription is not affected by the mutation.

## Introduction

Efficient protein translation requires an accurate biogenesis of the ribosomes, which consists of a complex series of processes [1], [2]. It begins with the transcription of rRNA precursors from the seven *rrn* operons which undergo nucleolytic processing by RNases to generate the mature *rrn* molecules [3]. The final steps of rRNA maturation occur in the early stages of protein synthesis by the newly-assembled ribosome [4], [5].

Transcription of the *rrn* genes is regulated mainly at the level of transcription initiation but further control involves an antitermination system which overcomes *rho*- dependent transcriptional terminators located along the *rrn* operon [6], [7]. This system prevents transcriptional polarity of the *rrn* operon and insures that all three RNA species (5, 16 and 23 S) are transcribed in equal amounts [8].

The formation of the transcription antitermination complex requires an RNA sequences known as “*nut* like sequences” which are located in the leader of the 16 S and in the spacer between the 16 S and the 23 S. These elements include three sequences: *boxA*, *boxB* and *boxC* which bind the cellular factors that construct the antitermination complex [9], [10]. These cellular factors include the Nus factors (factors also involved in the transcription antitermination of phage lambda [11]) and several ribosomal proteins [12], [13]. The factors facilitate the interaction between the *box* elements, RNAP and Rho and secure the transcription antitermination process [14]–[17].

The transcription antitermination process has two roles in addition to its primary function: to assist processing of the mature *rrn* transcript from the precursor state [18]–[21] and to modulate the transcription elongation rate [16], [22] thus ensuring the proper folding of the rRNA.

It is, therefore, clear that the transcriptional antitermination system is critical for several stages of ribosome biogenesis. Here we present evidence suggesting the involvement of a newly-analyzed protein YbeY in the transcription antitermination process.

YbeY is a 17 kD heat shock protein that belongs to the UPF0054 family and is highly conserved in bacteria [23]–[25]. Previous studies indicated that YbeY is important for translation and its absence results in production of impaired 30 S ribosomal subunits because of abnormal maturation of rRNA [26]–[28]. Recently [29] YbeY was shown to be a metallo endoribonuclease and with several functions including rRNA maturation and 70 S ribosome quality control. The results presented here indicate that YbeY has an additional role in transcriptional antitermination, that is critical for production of ribosomal subunits and could explain the defect in rRNA maturation.

## Results

### YbeY Affects the Transcription Antitermination of RNA

**Figure 1:**
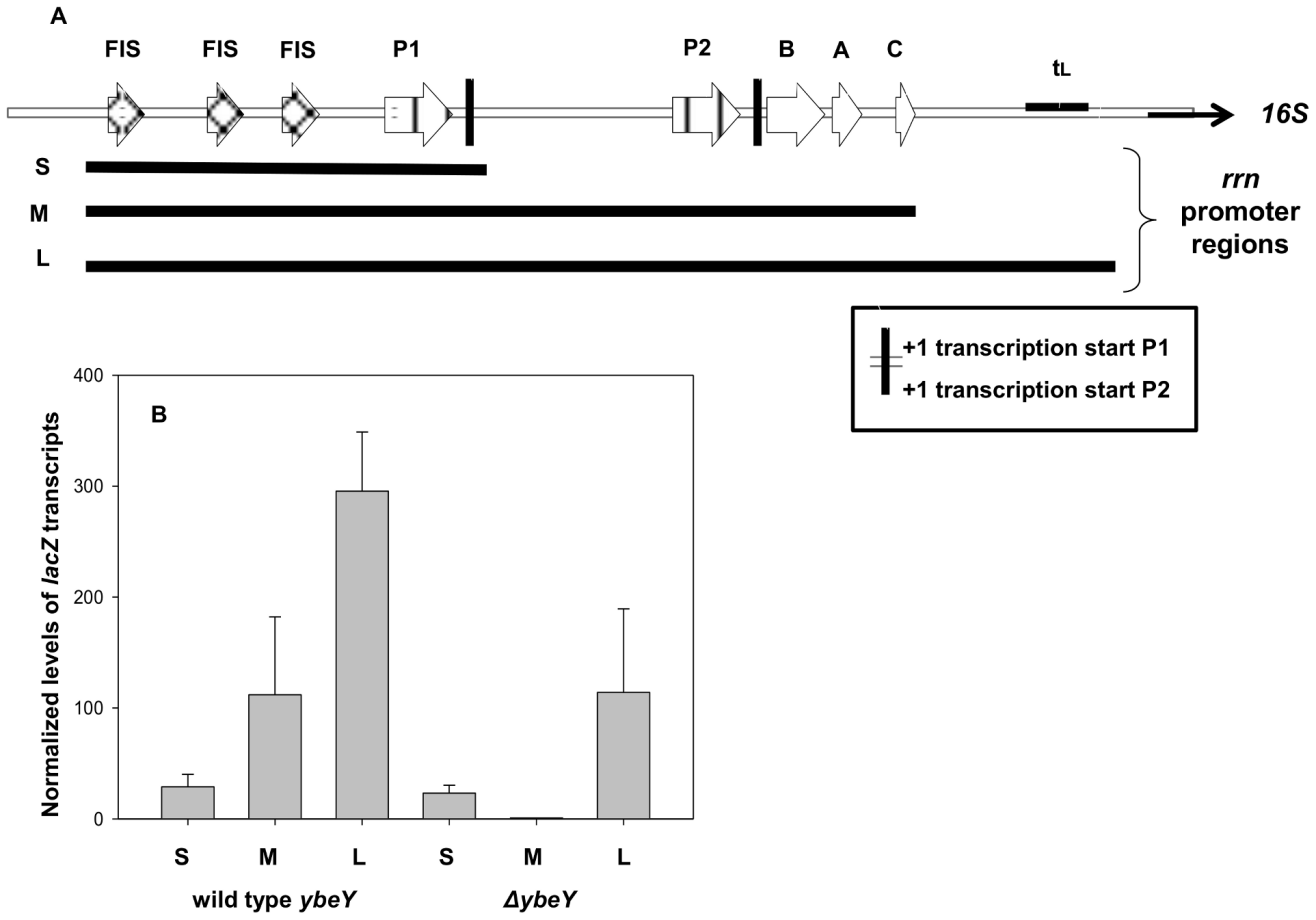
Transcript levels of *lacZ* transcribed from various *rrn* promoter regions. The pACYC plasmids were constructed so that they contained various *rrn* operon promoter regions fused upstream a promoterless *lacZ* gene, as described in Materials and Methods. The short promoter region of *rrn* contained only the P1 sequence of the *rrn* promoter (S = short), the second segment contained the P1, P2 and *nut* -like sequences (*boxA*, *boxB* and *boxC)* (M = medium length) and a longer segment contained the P1, P2, *nut* like sequences and t_L_ region of the *rrn* operon (L = long). All the promoter regions contained the FIS elements and other elements required for initiation of transcription. The pACYC plasmids were transformed into the *lacZ* and *ybeY, lacZ* deletion strains. (A) a schematic description of the three promoter regions which were fused to *lacZ*. The *rrn* sequence, the FIS elements, P1, P2, *nut* -like sequences and t_L_ region are marked. (B) Cultures of Δ*lacZ* (“wild type *ybeY*”) and Δ*lacZ*Δ*ybeY* (“Δ*ybeY*”) carrying the pACYC plasmids were grown in LB at 37°C and harvested at O.D_600_ = 0.45. RNA was extracted from these samples and analyzed by qRTPCR.

It has been shown that YbeY is essential for correct rRNA maturation, but the molecular basis of this effect has not been elucidated. One factor that could play a role in RNA maturation is the transcription antitermination process which is important for maintaining an optimal elongation rate for correct processing and folding of the RNA. In order to examine the possible effect of YbeY on the transcription antitermination process, we constructed a series of pACYC plasmids (NEW ENGLAND BioLabs) which contain three types of the *rrn* promoter regions fused upstream to a promoterless *lacZ* gene (Figure 1A). These promoter regions differ in respect to the presence of the antitermination “*nut*-like” sequences. The first *rrn* promoter region was short and contained only the P1 promoter sequence of the *rrn* promoter (S = short). The second *rrn* promoter contained the P1 and P2 promoters as well as the *nut* -like sequences (*boxA*, *boxB* and *boxC)* involved in transcription antitermination (M = medium length). The third and longest promoter region contained the P1 and P2 promoters, *nut* -like sequences and the t_L_ region of the *rrn* operon, which is a putative Rho-independent pausing site of transcription (L = long). All the regions also include FIS elements and additional sequences required for transcription initiation. In addition, the *lacZ* contains four Rho- dependent terminators. Using this experimental system it is possible to examine the affect of YbeY on transcription antitermination.

These plasmids were transformed into the wild type strain and the Δ*ybeY* strain, both containing a *lacZ* deletion (Δ*lacZ* ). The *lacZ* mRNA levels were measured by qRT PCR. These *lacZ* mRNA levels represent the levels of transcription from each promoter. We assumed that if YbeY is involved in transcription antitermination, then the *ybeY* deletion would not affect transcription from the short promoter region (S) but would alter transcription from the promoter regions which involve the transcription antitermination sequences, i.e., M and L.

Indeed, the results presented in Figure 1B indicate that transcription from the “S” promoter is not affected by the Δ*ybeY* mutation. In contrast, this mutation results in a significant reduction (100 fold) of transcription from the “M promoter” which contains the transcription antitermination sequences (“*nut* -like sequences”). The presence of the t_L_ region relieves some of the reduction: transcription from the “L promoter” is reduced only by 50% in the mutant (Figure 1B).

**Figure 2:**
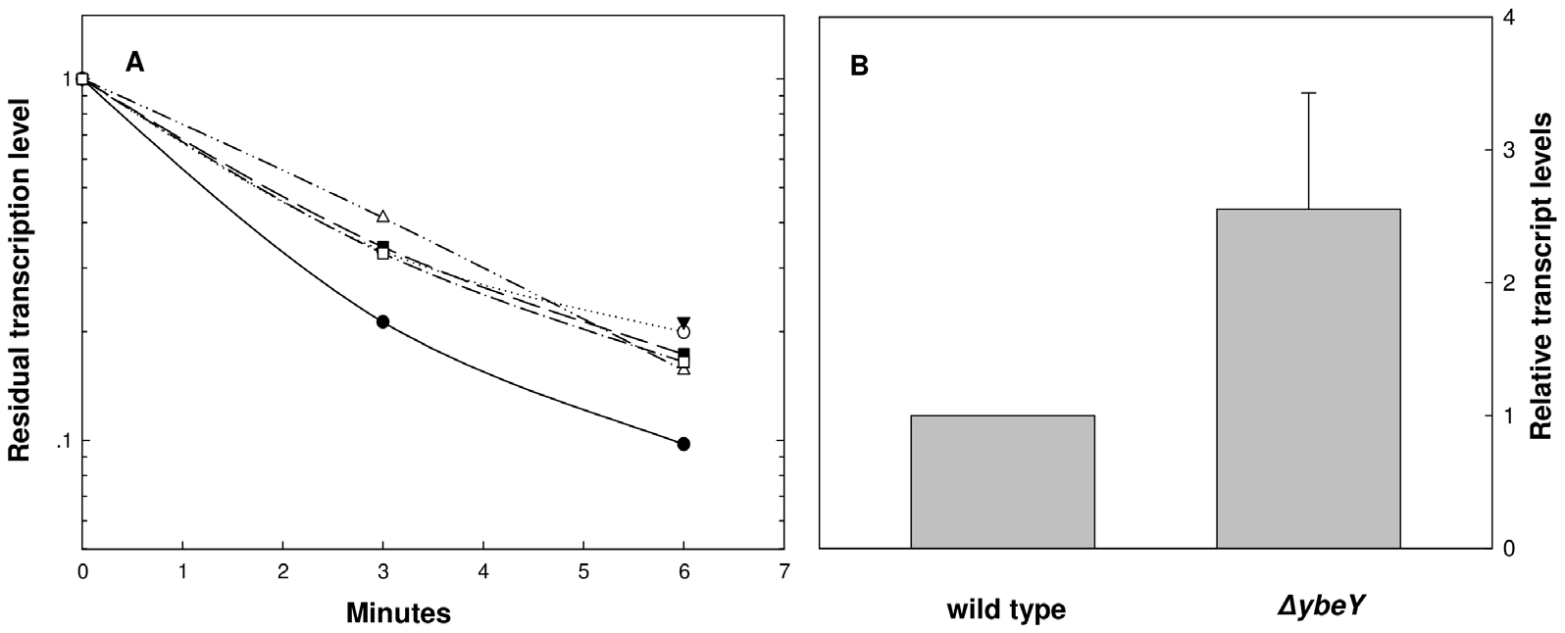
Stability and levels of RNA expressed from various *rrn* promoter regions in *ybeY* deletion mutant. (A) Stability of *lacZ* gene transcripts was determined by measuring its residual levels following addition of Rifampicin (200 µg/ml). Cultures and growth conditions were as described in Figure 1. All the bacteria were deleted for the chromosomal *lacZ* gene and transformed with pACYC plasmids carrying various *rrn* promoter sequences fused upstream to a promoterless *lacZ*. The plasmids carried the “S” region, “M” region, or “L” region. Rifampicin was added at time 0 and the cultures were harvested at 0, 3, 6 min. RNA was extracted and analyzed by qRTPCR. Bacteria with wild type *ybeY* gene and plasmid with “S” region (filled circles), with “M” region (open circles) and with “L” region (filled triangles). Δ*ybeY* mutant carrying a plasmid with “S” region (open triangles), with “M” region (filled squares) and with “L” region (opened squares). (B) Levels of RNA encoded from the region prior to the antitermination sequences. Cultures of wild type and Δ*ybeY* mutant were grown in LB at 37°C and harvested at O.D_600_ = 0.45. RNA was extracted from these samples and the levels of the transcripts encoded by the region before the antitermination sequences were quantified by qRTPCR from the primers described above.

The reduced levels of transcription from regions containing the transcription antitermination sequence presumably reflect the effect of the *ybeY* deletion on transcription antitermination. Alternatively, this reduction could also result from a reduced stability of transcripts produced from the “M promoter” region and the “L promoter” region in the Δ*ybeY* deletion strain. However, this latter possibility was excluded by experiments in which transcript stability was determined following the addition of the transcription inhibitor Rifampicin (Figure 2A). The results demonstrate that the deletion of *ybeY* does not result in decrease transcript stability, indicating that differential stability of transcripts is not the cause of the YbeY effect on transcription antitermination.

The reduction of *lacZ* transcripts from the “M” and “L” promoters correlates with the accumulation of short transcripts in the *ybeY* mutant as detected by qRT-PCR (Figure 2B) further supporting the role of YbeY in transcription antitermination. .

### Factors Participating in the Transcription Antitermination Process Compensate for the *ybeY* Deletion

**Figure 3:**
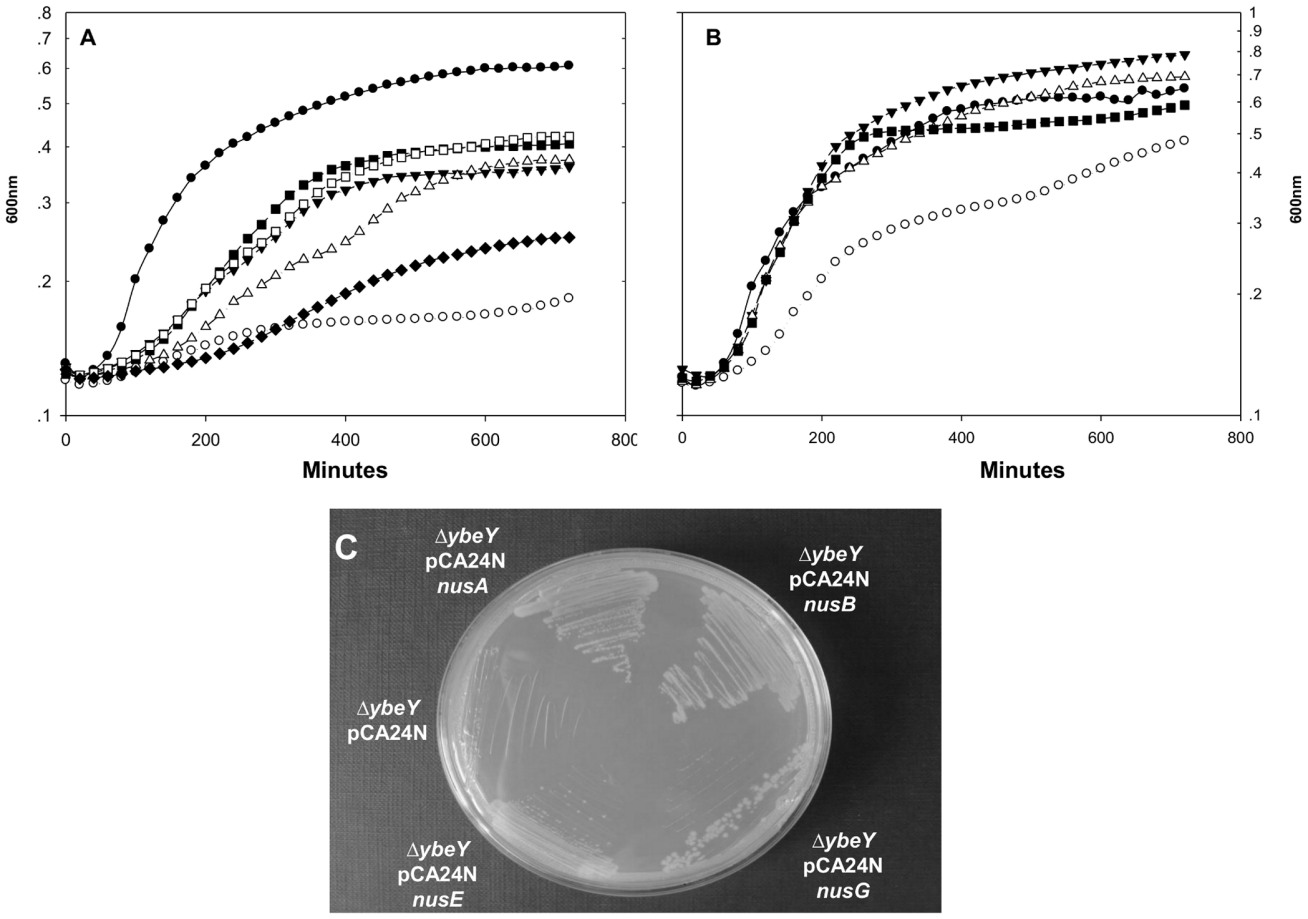
Effect of plasmids expressing transcriptional antitermination factors on growth of *ybeY* deletion mutant. Over-expression of NusA, NusB, NusG and NusE, was obtained by introducing multi-copy plasmids containing these genes cloned downstream to a *lacZ* promoter. These plasmids were transformed into the *ybeY* deletion mutant (A) and into the wild type strain (B). Cultures were grown overnight in LB medium at 30°C. They were then diluted to *A*600 of 0.04 in 2 ml wells (in a 24 well plate) containing 1 ml of LB medium supplemented with 0.1 mM IPTG for inducing the *lacZ* promoter. The cultures were transferred to 42°C and turbidity was measured at 600 nm for 12 hours. (A) wild type (filled circles), *ybeY* deletion mutant (open circles), Δ*ybeY* mutant carrying pCA24N *nusA (*filled triangles), Δ*ybeY* mutant carrying pCA24N *nusB* (open triangles), Δ*ybeY* mutant carrying pCA24N *nusE* (filled squares), Δ*ybeY* mutant carrying pCA24N *nusG* (opened squares) and Δ*ybeY* mutant carrying pCA24N empty vector (filled diamonds). (B) wild type (filled circles), wild type carrying pCA24N *nusA* (open circles), wild type carrying pCA24N *nusB* (filled triangles), wild type carrying pCA24N *nusE* (open triangles) and wild type carrying pCA24N *nusG* (filled squares). (C) Growth of Δ*ybeY* mutant carrying pCA24N encoding Nus genes on LB agar plates, supplemented with 0.1 mM IPTG, overnight at 42°C.

The results showing that YbeY is involved in the transcriptional antitermination process suggest the possibility that over-expression of factors participating in the transcription antitermination process could compensate for the effect of a *ybeY* deletion. Thus, we examined the effect of over-expression of several Nus factors on growth rate of the *ybeY* mutant. The experiment was carried out at 42°C, when the mutant has its most severe phenotype. The results indicate that, indeed, high cellular concentrations of NusA, NusB, NusE and NusG partially compensated for the *ybeY* mutation (Figure 3A and 3C), while they had virtually no effect in the wild type (Figure 3B).

## Discussion

YbeY is involved in ribosome biogenesis - in its absence ribosomes are defective and translation is reduced [26], [28], especially at elevated temperatures [27]. Here we show the involvement of YbeY in the transcriptional antitermination process of rRNA synthesis, which is critical for ribosome biogenesis. Thus, we show that YbeY is essential for *rrn* transcription of regions that contain the antitermination sequences (Figure 1). Transcription from the P1 promoter of *rrn* (S promoter) is unaffected by the absence of YbeY, but the transcription is almost abolished if the promoter region also contains the P2 and *nut* -like sequences which constitute the antitermination region (M).

The presence of t_L_ - the Rho-independent pause site [30] in the promoter region which contains the transcriptional antitermination site elevated the level of transcription - compare the effect of the deletion on transcription from the “M” promoter and the “L” promoter. The t_L_ is a conserved sequence in the leader region of rRNA and various deletions of the t_L_ results in rRNA transcription polarity [31]. It appears that t_L_ may assist a correct transcription antitermination process, and its presence enables the complex to be fully organized and stabilized prior to the transcription of the 16S rRNA. The lack of t_L_ may lead to premature termination during transcription. The effect of t_L_ on transcription can be seen in Fig. 1, as its presence stimualtes transcription in the wild type bacteria. Interestingly, the effect of t_L_ remains even in deletion mutants of *ybeY* where its presence increases the transcription level as well. Yet even in the presence of t_L_ there is still a 50% decrease in transcription of *rrn*. Such a decrease is probably sufficient to cause the phenotypes seen in the *ybeY* deletion mutants, as its effect may escalate into the major effect on ribosome biogenesis.

We assume that the reduced level of transcripts overlapping the transcriptional antitermination region in *ybeY* mutants results from the involvement of YbeY in the transcriptional antitermination process. However, it is also possible that this reduced level results from a lower stability of transcripts in the *ybeY* mutant. This possibility is ruled out by the experiment presented in Fig. 2, which shows that the stability of the M-transcripts (that contain the transcriptional antitermination sequence) is not affect by the *ybeY* deletion. In contranst, it should be mentioned that the stability of the short transcripts (from promoter S, that do not contain the transcriptional antitermination sequences) is affected by the *ybeY* mutation, but in the opposite directions. Thus, in the absence of YbeY the short transcripts are more stable. This result could be explained by the recent findings of Jacob et al [29] that attributed RNase activity to YbeY. This RNase may be more active on short transcripts, as these are not yet covered by the binding of various protein factors.

Further support for the effect of YbeY on the transcription antitermination of rRNA transcripts is obtained by the findings that the *ybeY* deletion mutation can be partially complemented by over-expression of the NUS factors involved in transcription antitermination (Figure 3). The overexpression of these NUS factors has virtually no effect on the wild type. Although there is no trivial explanation for these findings - that are highly significant and reproducible - we assume that these transcriptional factors affect the rate of transcription in the mutant by supporting the correct folding of the rRNA and thus improving rRNA maturation even in the absence of YbeY.

The results presented here show the importance of YbeY for ongoing rRNA transcription elongation. They are also compatible with the findings that immature 16S RNA accumulates in *ybeY* deletion mutants [28], as a correct transcription antitermination process is required for accurate maturation of rRNA. The maintenance of an optimal transcription elongation rate which allows the proper maturation and folding of the rRNA becomes difficult when the temperature is increased, as at the higher temperatures the rate of transcription increases. Therefore, the suggested involvement of YbeY in the transcription antitermination process and in assuring proper RNA maturation implies that its importance may increase with temperature. This assumption is compatible with the finding that the *ybeY* deletion has a stronger phenotype at high temperatures - the deletion mutant is temperature sensitive [27]. Moreover, YbeY is a conserved heat shock protein which is induced upon temperature shift-up, probably ensuring sufficient levels of this protein to satisfy the increased requirement upon shift to higher temperatures.

## Materials and Methods

### Bacterial Strains and Plasmids

*E. coli* MG1655 (ATCC 47076), a wild-type K-12 strain, was used in all experiments. The Δ*ybeY* deletion mutation was obtained as described [27]. The *lacZ* deletion [32] was introduced by the λRed system [33] to create wild type Δ*lacZ* or double mutants Δ*ybeY,* Δ*lacZ*. Plasmid pAC24N encoding Nus factors were transformed from the *E. coli* ASKA library [34] into *E. coli* MG1655 and its *ybeY* deletion strain.

### Growth Conditions

Unless otherwise stated, cultures were grown exponentially in LB broth (Difco) with aeration at 37°C to OD*600* = 0.45. The turbidity readings were performed using the Biotek Synergy EONTM reader, with 1 ml volume in 24 well plates.

### Construction of Plasmids

Plasmids (pACYC 184) encoding *rrn* promoter regions and fused upstream to a promoterless *lacZ* gene were constructed so that they contained three types of *rrn* operon promoter regions. The coding sequence of *lacZ* was PCR amplified using primers containing restriction sites for BamHI and HindIII. The digested PCR product was ligated into pACYC digested with the same restriction enzymes. Next, the promoter region of *rrnB* was PCR amplified using primers containing restriction sites for XbaI and HindIII. The digested PCR product was ligated into pACYC containing the *lacZ* product, digested with the same restriction enzymes. The pACYC plasmids encoding the *rrnB* promoter reign and *lacZ* were transformed into the *lacZ* and *ybeY lacZ* deletion strains. The primers used for plasmids construction are listed in Table 1.

**Table 1:** Primers used for plasmids construction.

Primer	Sequence
S Forward primer	5_ GCGTCTAGATGCGAATATTGCCTTTTGTA _-3
S Reverse primer	5_ GCGAAGCTTTGCCGTTGTTCCGTGTC _ -3
M Forward primer	5_ GCGTCTAGATGCGAATATTGCCTTTT _-3
M Reverse primer	5_ ATTAAGCTTACTTGGTATTCATTTTTCGT _-3
L Forward primer	5_ GCGTCTAGATGCGAATATTGCCTTTT _-3
L Reverse primer	5_ TAGAAGCTTTAAAAGTTTGACGCTCAA _-3
LacZ Forward primer	5_- GCGAAGCTTATGACCATGATTACGGATTCA _-3
LacZ Reverse primer	5_- TTGGGATCCTTATTTTTGACACCAGACCAAC _ -3

### RNA isolation

Total RNA was isolated using the RNA Protect and RNeasy kits (Qiagen) as described by the manufacturer and DNase I (Promega) was used to remove genomic DNA contamination.

### Quantification of Transcript Levels

Reverse transcription was carried out with one microgram of the DNase-treated total RNA using random hexamers (Promega) with ImPromII reverse transcriptase (Promega). Quantitative PCRs were performed using 250 nM of each gene-specific pair of primers in a 20 µl volume with 1 Sybr green PCR master mix (Applied Biosystems). Reactions were run on an ABI 7700 instrument (Applied Biosystems) using the following cycling parameters: 95°C for 10 min, 40 cycles of denaturation at 94°C for 15 second and extension at 60°C for 1 min.

For determining *lacZ* transcript levels the results were normalized to the *cat* levels in each sample, to account for possible deviations in *lacZ* levels due to changes in plasmid copy number. The primers used for quantifying the levels of *lacZ* and *cat* are listed in Table 2.

**Table 2:** Primers used for quantifying the level of transcripts in Quantitative PCR.

Gene	Primer	Sequence
*lacZ*	Forward primer	5_-CCTATCCCATTACGGTCAAT _-3
	Reverse primer	5_-CGTTGCACCACAGATGAAA_ -3
*cat*	Forward primer	5_AAGACGGTGAGCTGGTGAT_-3
	Reverse primer	5_TGCGAATATATGTGTAGAAACTG_-3

For determining the levels of the short transcripts (before the antitermination region) we used the following primers:

F: 5-_ TGACACGGAACAACGGCAAACACG_-3 and

R: 5-_ TGCATAATACGCCTTCCCGCTACA-_3.
